# Combined low-dose LiCl and LY294002 for the treatment of osteoporosis in ovariectomized rats

**DOI:** 10.1186/s13018-019-1210-1

**Published:** 2019-06-13

**Authors:** Jianhai Bai, Yier Xu, Yan Dieo, Guicai Sun

**Affiliations:** 1 Department of Ophthalmology, Taizhou Central Hospital (Taizhou University Hospital), 999 Donghaidadao St, Jiaojiang District, Taizhou, 318000 Zhejiang Province China; 2 School of Chemistry and Chemical Engineering, Harbin Institute of Technology, Harbin, China; 3 The Fourth Affiliated Hospital of Nanchang University, 133 Guangchangnan St., Xihu District, Nanchang, China

**Keywords:** NFATC1, Osteoporosis, Osteoblasts, Lithium chloride, LY294002

## Abstract

**Background:**

To provide a low-toxicity and high-efficacy clinical treatment for osteoporosis via a novel combination of LiCl and LY294002.

**Methods:**

The protein levels of p-AKT, AKT, p-GSK3β, GSK3β, β-catenin, p-β-catenin, and NFATC1 were measured in osteoblasts and osteoclasts by Western blot. ALP activity and TRACP activity were measured using the corresponding kit. The levels of BALP, PINP, CTX, and TRACP-5b were determined in accordance with the requirements of the ELISA kits. Microstructural analysis was performed on the left distal femur using microcomputed tomography.

**Results:**

Treatment with the combination of LiCl and LY294002 led to a markedly increased osteoblast activity but significantly decreased osteoclast differentiation and bone absorption capacity compared with the treatment with LiCl or LY294002 alone (*P* < 0.01). In serum, the low-dose combination of LiCl and LY294002 significantly enhanced BALP levels (*P* < 0.01) and significantly decreased PINP, TRACP-5b, and CTX levels (*P* < 0.01) compared with the application of either drug alone.

**Conclusions:**

This study indicates that drug combinations directed at multiple targets could be used for osteoporosis treatment by promoting osteoblast proliferation and inhibiting differentiation with high efficiency.

## Background

Bone is regenerated continuously via a process known as remodeling. Bone remodeling includes formation and resorption processes mediated by osteoblasts and osteoclasts, respectively [1]. The balance between bone formation and absorption is disrupted with age, and both the mechanical strength and mass of bone decrease because of the loss of bone cell function through bone tissue microstructural deterioration, decreased trace elements and salt levels, and increased osteoblast apoptosis, all of which increase bone porosity and are considered symptoms of osteoporosis [2, 3]. Currently, the main treatment for osteoporosis is stimulating bone formation or inhibiting bone resorption [4]. However, chemicals that promote bone formation exert their effects slowly, whereas those that inhibit bone resorption are toxic and have negative side effects [5, 6], thus complicating the treatment. Therefore, the combined use of two or more drugs for the treatment of osteoporosis to reduce toxicity and increase the efficacy is one strategy for improving osteoporosis treatment.

The Wnt and AKT signal transduction pathways can promote osteoblast proliferation and inhibit osteoclast differentiation [7, 8]. It has been reported that Wnt proteins, irrespective of their ability to stimulate canonical Wnt signaling, prolong the survival of osteoblasts and uncommitted osteoblast progenitors via activation of the Src/ERK and phosphatidylinositol 3-kinase (PI3K)/AKT signaling cascades [9]. Ariokaet et al. [10] studied a lithium chloride (LiCl) intervention using osteoblasts and osteoclasts and observed that the local injection of LiCl promoted osteoblast proliferation and inhibited osteoclast differentiation by inhibiting glycogen synthase kinase 3-β (GSK3β), thereby improving local bone regenerative capacity. Moon et al. [11] determined that LY294002 can suppress AKT activity in osteoclasts by affecting PI3K, thus inhibiting osteoclast differentiation. Therefore, the AKT/GSK3β/β-catenin/NFATC1 signaling pathway not only increases osteoblast proliferation and differentiation [12] but also regulates osteoclast differentiation and plays a vital role in bone metabolism [13].

In this study, the AKT/GSK3β/β-catenin/NFATC1 signaling pathway in osteoblasts and osteoclasts was first examined in vitro to determine whether the inhibitory effect of the low-dose combination of LiCl and LY294002 on the AKT/GSK3β/β-catenin/NFATC1 signaling pathway was greater than the effects of either of the two drugs alone. Second, in vitro bone formation and resorption were investigated to evaluate whether the low-dose combination of LiCl and LY294002 could better promote the bone formation of osteoblasts and inhibit the bone resorption of osteoclasts compared with either of the two drugs alone. Third, the products of bone metabolism in rats were detected to verify whether the low-dose combination of LiCl and LY294002 more strongly promoted bone formation and inhibited bone resorption in vivo than either of the two drugs alone. Finally, the spongy region of the distal rat femur was analyzed using microcomputed tomography (μ-CT), which provided further validation of whether the LiCl and LY294002 low-dose combination therapy was more efficacious in treating osteoporosis than either of the two drugs applied alone.

## Methods

This study was performed in strict accordance with the recommendations of the Guide for the Care and Use of Laboratory Animals of the Chinese Society of Experimental Animals. The protocol was approved by the Medical Ethics Review Committee of Harbin Medical University (Ref No. 16/032/HMU). All surgeries were performed under sodium pentobarbital anesthesia, and all efforts were made to minimize animal suffering. The researchers optimized the experimental procedures and treated the animals according to the ethical guidelines; furthermore, the indices observed in the experiments were adjusted to ensure animal welfare.

### Experimental animals and groups

Thirty-five Sprague-Dawley (SD) rats weighing 220 ± 10 g at 6 months of age were fed in an environment maintained at 20–24 °C and 45–60% humidity under a 12-h light/dark cycle. The ovaries were surgically removed via lower abdomen incision from all experimental animals except those in the sham operation group. Femur injections by X-ray guidance were performed on the 1st and 30th days after surgery. The control and model groups were administered in the same volume of phosphate-buffered saline (PBS), whereas the other three groups were injected with LiCl (10 mM), LY294002 (using 0.1–0.3% dimethylsulfoxide (DMSO) as a vehicle control and dissolved at 0.1%, 10 μM), or LiCl (5 mM) + LY294002 (5 μM) [14, 15]. After 8 weeks, all rats were anesthetized and sacrificed.

All instruments were sterilized by epoxy ethane prior to surgery. The rats were fasted between 12 and 16 h prior to surgery, and 12 h of fasting is considered the optimal time. The rats were anesthetized prior to surgery by intraperitoneal injection of 10% chloral hydrate solution at a dose of 0.3 mL/100 g and were placed in lateral position. The rats were shaved, and a subcostal approach was used by which a 10-mm incision was made at the 12th rib, followed by blunt dissection of the subcutaneous tissue and the artery until the ovary (red, mulberry-like) was fully exposed. The ovarian tissue connects to the uterus. Silk thread was used to ligate the uterus, which is connected to the ovary and separates the ovary from the uterus. Silk thread was then used to ligate the adipose mass under the ovary to reduce bleeding. The ovary was completely removed, and the adipose mass was returned to the abdominal cavity. Finally, the muscles, fascia, and skin were sutured layer by layer [16]. Rats in the sham-operated group underwent the same procedures to open the abdomen, but only a small amount of retroperitoneal fat tissue was resected instead of the ovary.

For the in vivo experiments, the animals were divided into the following five groups (seven rats in each group): vehicle, OVX, LY294002, LiCl, and LY294002 + LiCl groups. The in vitro experiments consisted of four groups: vehicle (*n* = 3), LY294002 (*n* = 3), LiCl (*n* = 3), and LiCl + LY294002 (*n* = 3) groups. The concentrations of intervention drugs were the same for the in vitro and in vivo experiments.

### Materials and antibodies

LiCl was purchased from NacalaiTesque (Kyoto, Japan). α-Minimum essential medium (α-MEM) was purchased from GIBCO BRL (Grand Island, NY, USA). α-MEM for basal culture was purchased from HyClone (cat: SH30023.01B). Fetal bovine serum (FBS) was purchased from GIBCO (cat: 16400-044). Human recombinant receptor activator of nuclear factor-B ligand (RANKL, cat: 400-30, lot: 0209437) and macrophage colony-stimulating factor (M-CSF, cat: 400-28, lot 0310418) were purchased from PeproTech. Secondary antibodies were purchased from Beijing Xia Si Biotechnology Co., Ltd., China (Prospec, cat: SV30010). Antibodies against GSK3β (Ab-9; cat: A7098), GSK3 (cat: B7098), AKT (Ab-124; cat: B0407), AKT (phospho-Ser473; cat: A7005), matrix metalloproteinase 14 (cat: CO266), β-catenin (Ab-33/37; cat: B7022-1), and β-catenin (phospho-Ser33 cat: A7022) were purchased from Assay Biotech. Goat anti-mouse horseradish peroxidase-conjugated IgG and goat anti-rabbit horseradish peroxidase-conjugated IgG were purchased from Nanjing Biological (cat: Abmart M21001 L) and Shanghai Biology (cat: HA1001), respectively. An alkaline phosphatase (ALP) test kit was purchased from the Nanjing Jiancheng Bioengineering Institute, China (A059-2). A bicinchoninic acid (BCA) test kit was purchased from the Shanghai Biyuntian Company (cat: p0012).

### Cell culture

#### Culture of osteoblasts

Primary osteoblasts were harvested from the calvariae of 3-day-old rats and washed with PBS. After splitting the muscle, the bone was cleaned 2–3 times by pipetting with a cold PBS solution containing penicillin/streptomycin (PS). Bone fragments 1 mm in length, width, and depth were created using a dental bur; the bone samples were placed in centrifuge tubes filled with 0.1% trypsin and then placed in a shaking water bath (120 cycles/min) for 20 min. Then, the calvariae were placed in 50-mL centrifuge tubes and incubated twice with 0.2% collagenase for 60 min at 37 °C in a shaking water bath (120 cycles/min). The reaction was terminated by the addition of growth medium and repeated pipetting, and the bone fragments were removed using a 200-mesh strainer. The filtrate was collected and centrifuged at 1200×*g* for 8 min at 4 °C, and then, the supernatant was discarded. The cells were resuspended in α-MEM with 10% FBS and 1% PS (2 mL/well) and incubated for 2 days with 5% CO_2_ at 37 °C. The medium was changed every 3 days. Cell morphology and growth were observed by inverted microscopy. These cells were counted and used in different experimental studies [17].

#### Primary culture of rat osteoclasts

The femora and tibiae of 5-week-old, male SD rats were shaved and washed with culture fluid. Osteoclast generation was achieved using primary cultures of rat bone marrow-derived macrophages. The cells were maintained in α-MEM supplemented with 10% FBS and 1% PS in a humidified atmosphere with 5% CO_2_ at 37 °C for 24 h. The nonadherent cells were then transferred to new plates and cultured in α-MEM with 25 ng/mL M-CSF + 50 ng/mL RANKL to observe the formation of osteoclasts [18].

### Western blot analysis

According to the instructions provided by the Bicinchoninic Acid (BCA) Kit (Boster, Wuhan, China), the extracted upper protein after centrifugation of cell sample was boiled in a loading buffer (30 μg/well) at 95 °C for a total of 10 min. Protein samples were separated using a 12% gel via electrophoresis and then transferred to a polyvinylidene difluoride membrane using a semidry transfer system (Millipore, Bedford, MA, USA). After 2 h incubation at room temperature with 5% skim milk powder, the primary antibody solution was added to the membrane and kept at 4 °C overnight; the membrane was then washed 5 times with TBS-T buffer for 10 min each and incubated for 1 h at room temperature. An enhanced chemiluminescence gel imaging system was used to capture images for analysis [19].

### Alizarin red staining

To examine the matrix mineralization of osteoblasts, cells were exposed to osteoblast induction medium containing 50 μg/mL vitamin C and 10 mM β-glycerophosphate. After the osteoblasts were incubated for 21 days, the supernatant was removed, and the cells were rinsed with PBS 3 times. The cells were then immobilized in situ for 10 min with 95% ethanol, rinsed twice with distilled water, stained with Alizarin red for 30 min, and rinsed twice with distilled water to allow the observation of red mineralized nodules. Cells in each group were observed by microscopy and imaged. Then, chlorinated hexadecane at room temperature was added to the cells, and the cells were allowed to stand for 30 min. The optical density of the resulting supernatant was measured at a wavelength of 562 nm using a spectrophotometer [20].

### Bone pit experiments

A 250-μL aliquot of α-MEM was added to each well of a 96-well hydroxyapatite (HA)-coated plate (Corning, Corning, NY, USA) and incubated for 2 days in 5% CO_2_ at 37 °C; subsequently, noninduced osteoclast precursors were added to the fixed cells and incubated for 2 days (hereafter in induction medium supplemented with 10% FBS, 1% 25 ng/mL M-CSF, and 50 ng/mL RANKL mycillin) in 5% CO_2_ at 37 °C. Cells were treated with or without various concentrations of LiCl, LY294002, or their combination for 14 days. Media Cybernetics software was used to analyze the images (Silver Spring, MD, USA) [9].

### ALP activity assay

The purpose of this part of the experiment was to prove that a combination of drugs could improve the activity of osteoblasts. Logarithmic phase rat osteoblasts were collected, cultured for 72 h, and then washed 3 times with PBS. The cells were recollected after repeated freezing in liquid nitrogen and thawing. ALP activity was measured using an ALP Test Kit (A059-2). The protein concentration was determined using a BCA Protein Assay Kit. The absorbance of the tubes from each group was determined using a spectrophotometer. The ALP enzymatic activity was calculated following formula (1):

$$ \mathrm{ALP}\ \mathrm{activity}\ \left(\mathrm{king}\ \mathrm{unit}/\mathrm{gprot}\right)=\left(\mathrm{optical}\ \mathrm{density}\ \left(\mathrm{sample}\right)-\mathrm{optical}\ \mathrm{density}\ \left(\mathrm{blank}\right)\right)/\left(\mathrm{optical}\ \mathrm{density}\ \left(\mathrm{standard}\right)-\mathrm{optical}\ \mathrm{density}\ \left(\mathrm{blank}\right)\right)\times 0.1\;\mathrm{mg}/\mathrm{mL}\ \left(\mathrm{standard}\ \mathrm{concentration}\right)/\mathrm{protein}\ \mathrm{concentration}\ \mathrm{of}\ \mathrm{the}\ \mathrm{sample}\ \left(\mathrm{gprot}/\mathrm{mL}\right). $$

### TRACP assay

The aim of this study was to evaluate the osteoclast activity using serum TRACP 5b, which is generally used in the clinic as a specific and sensitive marker of bone resorption. Logarithmic phase osteoclasts were collected, cultured for 72 h, and then washed with PBS 3 times. The cells were recollected after repeated freezing in liquid nitrogen and thawing. TRACP activity was measured using a TRACP Assay Kit (A058-2). Protein concentration was measured using a BCA Protein Assay Kit. The absorbance from the tubes in each group was determined using a spectrophotometer. Serum TRACP (STRACP) enzymatic activity was calculated following formula (2):

$$ \mathrm{STRACP}\ \mathrm{activity}\ \left(\mathrm{king}\ \mathrm{unit}/\mathrm{gprot}\right)=\left(\mathrm{optical}\ \mathrm{density}\ \left(\mathrm{sample}\right)-\mathrm{optical}\ \mathrm{density}\ \left(\mathrm{blank}\right)\right)/\left(\mathrm{optical}\ \mathrm{density}\ \left(\mathrm{standard}\right)-\mathrm{optical}\ \mathrm{density}\ \left(\mathrm{blank}\right)\right)\times 0.1\;\mathrm{mg}/\mathrm{mL}\ \left(\mathrm{standard}\ \mathrm{concentration}\right)/\mathrm{protein}\ \mathrm{concentration}\ \mathrm{of}\ \mathrm{the}\ \mathrm{sample}\ \left(\mathrm{gprot}/\mathrm{mL}\right). $$

### Determination of serum bone marker levels by ELISA

After abdominal anesthesia, the blood from the abdominal aorta of each group was collected and centrifuged at 3500×*g* for 15 min at 4 °C to obtain the serum. The levels of BALP, PINP, CTX, and TRACP-5b were determined in accordance with the instructions of the ELISA kits. The above methods were conducted in strict accordance with the manufacturer’s instructions.

### CT imaging

Microstructural analysis was performed on the left distal femur using μ-CT (CT 40, The Medical Company, Switzerland). The femoral alignment was perpendicular to the scanning axis. Scanning was conducted at 55 kVp with 10.5-μm slices at a resolution of 16 μm/pixel. The trabecular bone at the distal end of the femur was identified with a semiautomatically drawn contour in each two-dimensional (2D) section. The segmentation parameters were fixed at sigma = 0.5, support = 1.0, and threshold = 245. The region of interest (ROI) was defined as 2.1 cm below the distal femoral growth plate, which was determined using 200 serial sections. From the 3D reconstructed image, the structural parameters of the ROI were derived using the image analysis program of the μ-CT workstation. The direct model parameters were analyzed, including bone mineral density (BMD), structural model index (SMI), connectivity density (COD), bone surface to volume ratio (BS/BV), bone volume over total volume (BV/TV), trabecular number (Tb.N), trabecular thickness (Tb.Th), and trabecular separation (Tb.Sp).

### Statistical analysis

All data are shown as the mean ± standard error of the mean (SEM) and were analyzed by factorial ANOVA using SPSS13.0 (SPSS Inc., Chicago, IL). Significance was defined as *P < 0.05, **P < 0.01, or ***P < 0.001.

## Results

AKT/GSK3β/β-catenin/NFATC1 signaling has previously been shown to increase osteoblast proliferation and differentiation and to regulate osteoclast differentiation. To ascertain the biological relevance in the current study, we first investigated the effects of combining LiCl and LY294002 on this signaling pathway and the corresponding role in bone metabolism.

### Effects of LiCl and LY294002 alone and in combination on the AKT/GSK3β/β-catenin/NFATC1 signaling pathway in osteoblasts and osteoclasts

**Fig. 1:**
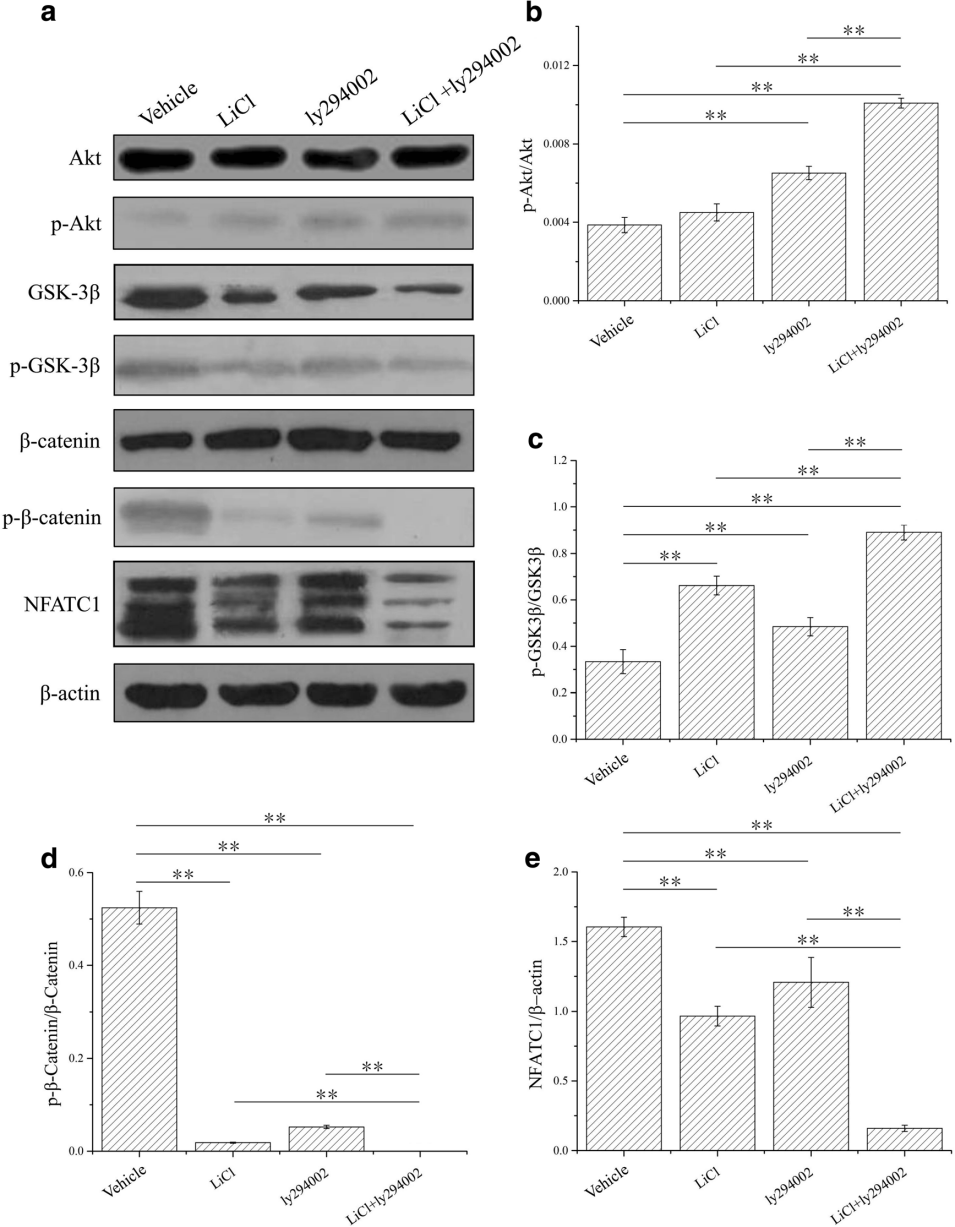
Western blot results for AKT/GSK3β/β-catenin/NFATC1 signaling in osteoblasts. **a** The results obtained from Western blot analyses indicate that the protein expression levels of p-AKT, GSK-3β, p-GSK-3β, p-β-catenin, and NFATC1 were significantly decreased and that of p-AKT was significantly increased in the samples treated with LiCl [cells cultured in α-MEM with LiCl (10 mM)], LY294002 [cells cultured in α-MEM with LY294002 (10 μM)], and LiCl + LY294002 [cells cultured in α-MEM with LiCl (5 mM) + LY294002 (5 μM)] compared with the samples treated with vehicle (cells cultured in α-MEM). **b** At 30 min, the ratio of p-AKT/AKT in cells cultured in LY294002 and LiCl + LY294002 was found to have significantly increased (**P < 0.01; *n* = 3). **c** At 30 min, the ratio of p-GSK3β/GSK3β in cells cultured in LiCl, LY294002, and LiCl + LY294002 was found to have significantly increased (**P < 0.01; *n* = 3). **d** At 30 min, the ratio of p-β-catenin/β-catenin in cells cultured in LiCl, LY294002, and LiCl + LY294002 was found to have significantly decreased (**P < 0.01; *n* = 3). **e** At 30 min, the ratio of NFATC1/β-actin in cells cultured in LiCl, LY294002, and LiCl + LY294002 was found to have significantly decreased (**P < 0.01; *n* = 3)

**Fig. 2:**
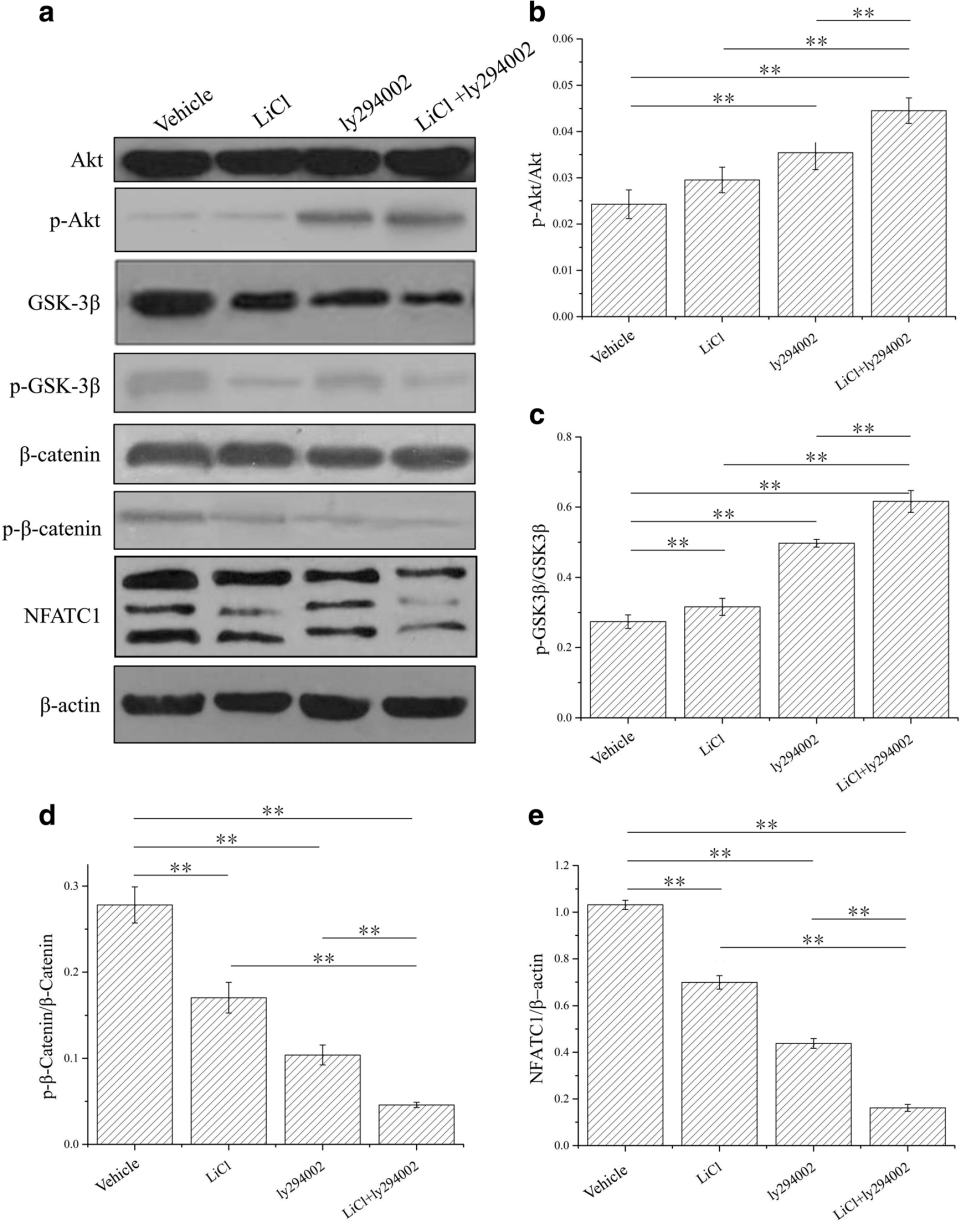
Western blot results for AKT/GSK3β/β-catenin/NFATC1 signaling in osteoclasts. **a** The results obtained from Western blot analyses indicate that the protein expression levels of p-AKT, GSK-3β, p-GSK-3β, p-β-catenin, and NFATC1 were significantly decreased and that of p-AKT was significantly increased in the samples treated with LiCl [cells cultured in α-MEM with LiCl (10 mM)], LY294002 [cells cultured in α-MEM with LY294002 (10 μM)], and LiCl + LY294002 [cells cultured in α-MEM with LiCl (5 mM) + LY294002 (5 μM)] compared with the control samples (cells cultured in α-MEM). **b** At 30 min, the ratio of p-AKT/AKT in cells cultured in LY294002 and LiCl + LY294002 was found to have significantly increased (**P < 0.01; *n* = 3). **c** At 30 min, the ratio of p-GSK3β/GSK3β in cells cultured in LiCl, LY294002, and LiCl + LY294002 was found to have significantly increased (**P < 0.01; *n* = 3). **d** At 30 min, the ratio of p-β-catenin/β-catenin in cells cultured in LiCl, LY294002, and LiCl + LY294002 was found to have significantly decreased (**P < 0.01; *n* = 3). **e** At 30 min, the ratio of NFATC1/β-actin in cells cultured in LiCl, LY294002, and LiCl + LY294002 was found to have significantly decreased (**P < 0.01; *n* = 3)

Figure 1a shows the alterations in AKT/GSK3β/β-catenin/NFATC1 signaling in osteoblasts after treatment with LiCl or LY294002 alone or treatment with the low-dose combination of LiCl and LY294002. After the administration of LiCl or LY294002 alone, the ratios of p-Akt/Akt and β-GSK3β/GSK3β protein increased while the ratios of p-β-catenin/β-catenin and NFATC1/β-actin protein decreased in osteoblasts. Interestingly, after administration of the low-dose combination of LiCl and LY294002, the same trend in the ratios became more evident (Fig. 1b–e). Comparatively, treatment with LiCl, LY294002, or their low-dose combination had the reverse effect on the ratios of protein content in osteoclasts (Fig. 2). These results indicate that the low-dose combination of LiCl and LY294002 exerts a greater inhibitory effect on AKT/GSK3β/β-catenin/NFATC1 signaling in osteoblasts and osteoclasts than either of the two drugs alone.

### Effects of LiCl, LY294002, and LiCl + LY294002 on mineralized nodule formation and ALP activity in osteoblasts

**Fig. 3:**
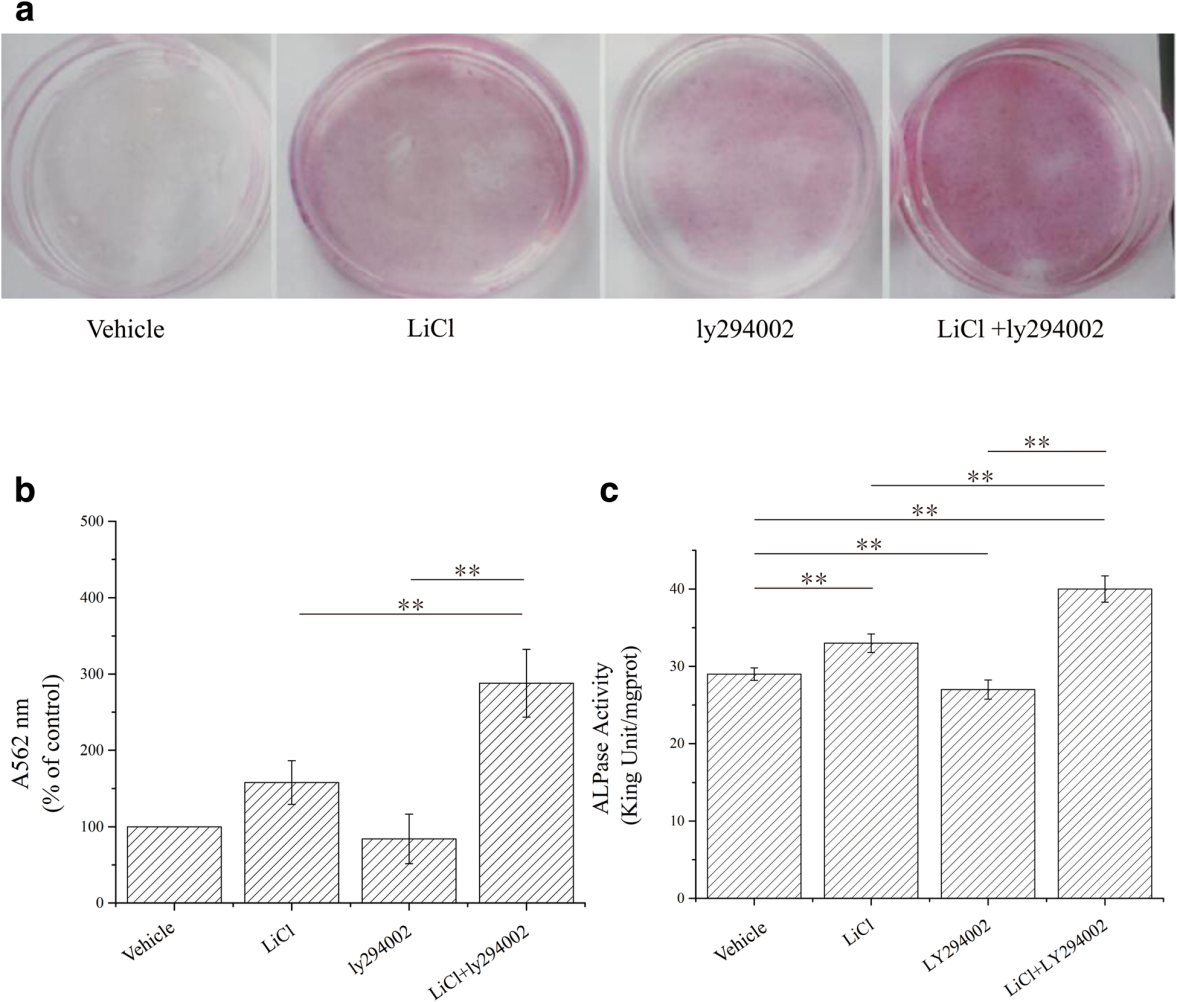
Mineralized nodules as visualized by Alizarin red staining and ALP activity in osteoblasts. **a** A total of 1.1 × 10^4^ osteoblasts per square centimeter were cultured in a 35-mm dish with either α-MEM, α-MEM with LiCl (10 mM), α-MEM with LY294002 (10 μM), or α-MEM with LiCl (5 mM) + LY294002 (5 μM) for 21 days (*n* = 3). **b** Quantitative results from Alizarin red staining of rat osteoblasts in each group. The counts of osteoblasts treated with LiCl + Ly294002 were significantly increased (**P < 0.01; *n* = 3). **c** ALP activity in osteoblasts was determined by a kit. The results are expressed as specific activity (king unit/mgprot) of osteoblasts cultured for 21 days (**P < 0.01; *n* = 3)

**Fig. 4:**
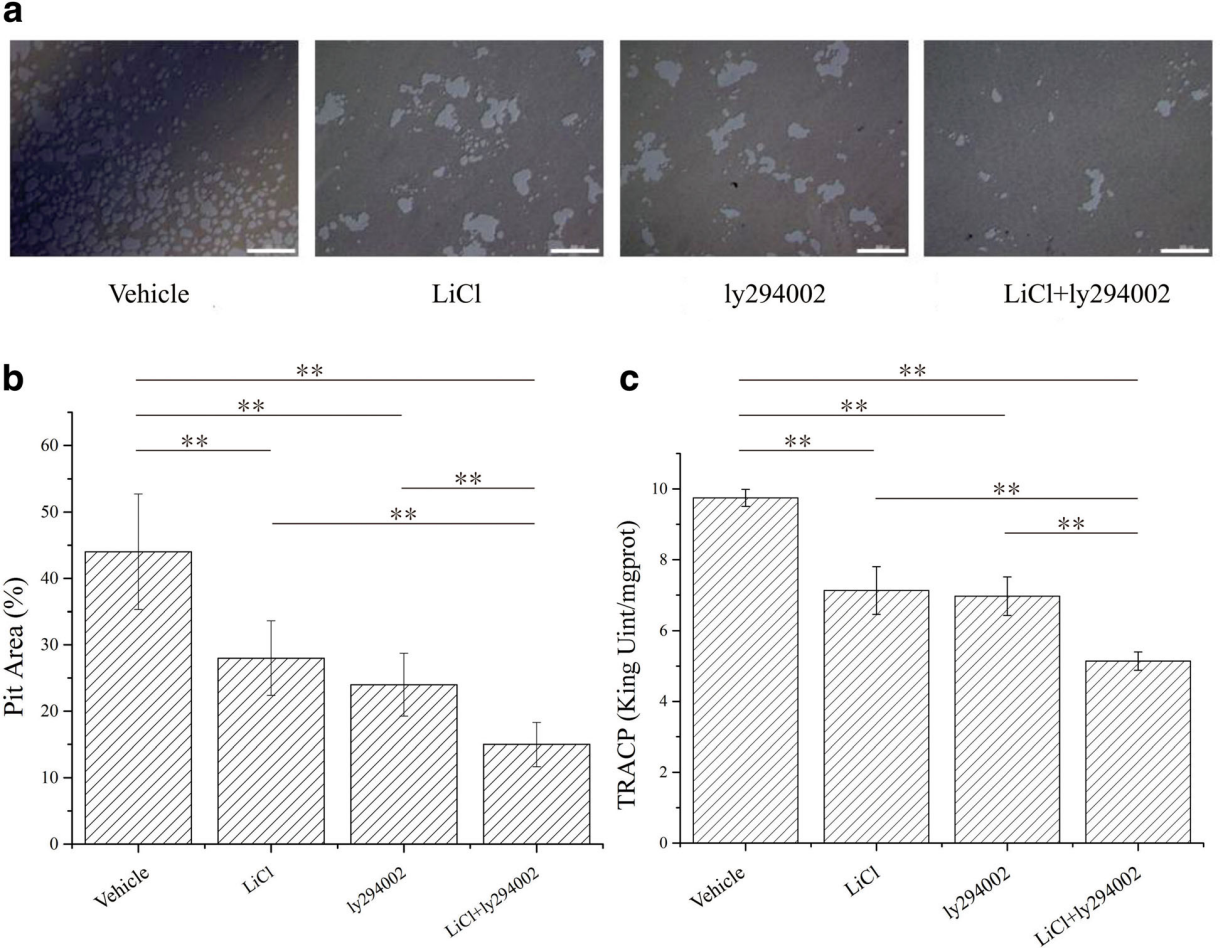
Effects of LiCl, LY294002, and LiCl + LY294002 on bone resorption and TRAP activity in osteoclasts. Control: cells were cultured in α-MEM. LiCl: cells were cultured in α-MEM with LiCl (10 mM). LY294002: cells were cultured in α-MEM with LY294002 (10 μM). LiCl + LY294002: cells were cultured in α-MEM with LiCl (5 mM) + LY294002 (5 μM). **a** Images showing osteoclasts in bone slices after 14 days of culture; scale bar = 200 μM. **b** Statistics of the pit area fragment (**P < 0.01; *n* = 3). **c** TRAP activity in osteoblasts was determined using a kit. The results are expressed as specific activity (king unit/mgprot) of osteoblasts cultured for 21 days (**P < 0.01; *n* = 3)

Whether the low-dose combination of LiCl and LY294002 could affect the bone formation and resorption capabilities of osteoclasts was investigated in vitro. In addition, the effects of LiCl, LY294002, or their low-dose combination on osteoblastic mineralized nodule formation, osteoclastic bone resorption, and functional marker levels in the two cell types were evaluated. The formation of mineralized nodules can be used to evaluate the bone formation ability of osteoblasts, and ALP is a good marker of osteoblast activity [21]. The mineralized nodule formation and ALP activity of osteoblasts treated with LiCl, LY294002, or their low-dose combination are shown in Fig. 3. LiCl promoted both mineralized nodule formation and ALP activity in osteoblasts. However, the low-dose combination of LiCl and LY294002 significantly increased the number of mineralized nodules formed and the ALP activity of osteoblasts (Fig. 3b, c). Bone pit depth can be used to evaluate the bone resorption ability of osteoclasts, and TRACP is a good marker of osteoclast activity [22], as its activity reflects the degradation ability of osteoclasts. The osteoclastic bone resorption capacity and TRACP activity in response to LiCl, LY294002, and their low-dose combination are shown in Fig. 4. Both LiCl and LY294002 alone reduced the bone resorption area and inhibited the activity of TRACP in osteoclasts; however, the bone resorption area decreased significantly after the administration of the low-dose combination of LiCl and LY294002 (Fig. 4b), and the TRACP activity of osteoclasts was significantly lower than that resulting from the administration of LiCl or LY294002 alone (Fig. 4c). These in vitro results show that the combined application of low-dose LiCl and LY294002 can promote bone formation and inhibit bone resorption more than the treatment with LiCl or LY294002 alone.

### Effects of LiCl and LY294002 and their combined application on serum BALP, PINP, TRACP-5b, and CTX levels in rats

**Fig. 5:**
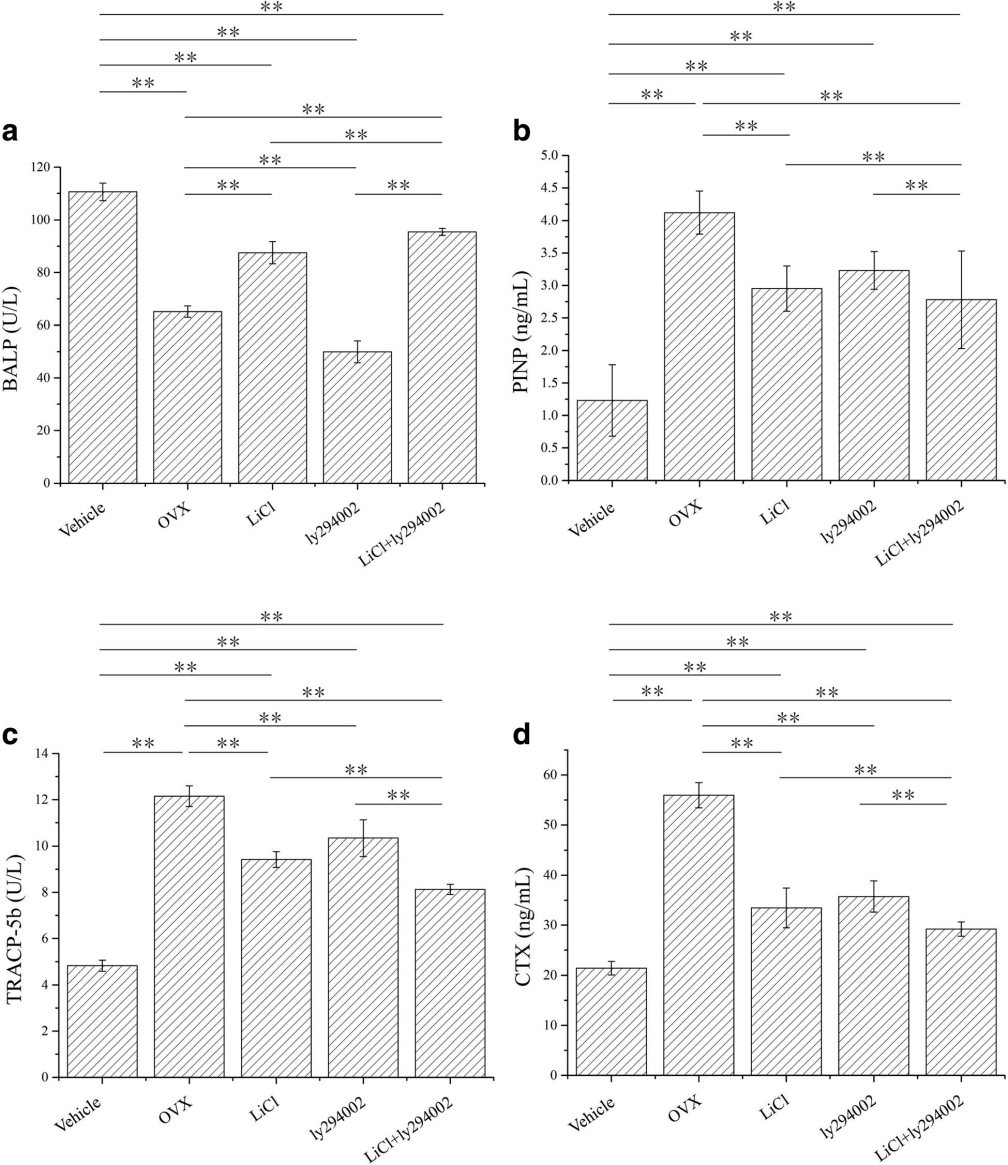
Effects of LiCl, LY294002, and their combined application on BALP, PINP, TRACP-5b, and CTX in the serum of OVX rats (*n* = 3). Control: rats were subjected to sham surgery. OVX: rats were ovariectomized. LiCl: ovariectomized rats were injected with LiCl (10 mM). LY294002: ovariectomized rats were injected with LY294002 (10 μM). LiCl + LY294002: ovariectomized rats were injected with LiCl (5 mM) + LY294002 (5 μM). The serum was separated to measure the content of bone alkaline phosphatase (BALP) activity (**a**), the content of PION (**b**), the tartrate-resistant acid phosphatase isoform 5b (TRAP5b) activity (**c**), and the content of CTX (**d**). *P < 0.05 and **P < 0.01 denote statistically significant differences

To further verify that the low-dose combination of LiCl and LY294002 was more effective than applying either of the two drugs individually and to determine whether the low-dose combination of LiCl and LY294002 exerted an anti-osteoporotic effect by promoting bone formation and inhibiting bone absorption, serum BALP, PINP, TRACP-5b, and CTX levels were detected in ovariectomized SD rats. Figure 5 shows that in serum, the level of the bone morphogenetic metabolite BALP decreased, whereas the levels of PINP, TRACP-5, and CTX increased compared with the vehicle, and the trend was opposite to OVX. Compared with the administration of LiCl or LY294002 alone, the low-dose combination of LiCl and LY294002 significantly increased the BALP serum content (Fig. 5a). Additionally, the combination therapy significantly reduced the serum levels of PINP TRACP-5b and CTX (Fig. 5b–d). These results suggest that the low-dose combination of LiCl with LY294002 exhibits anti-osteoporotic activity by promoting bone formation and inhibiting bone resorption and that the effect of the combined treatment was greater than that of the two compounds alone.

### Effects of LiCl, LY294002, and LiCl + LY294002 on the femur trabecular bone

**Fig. 6:**
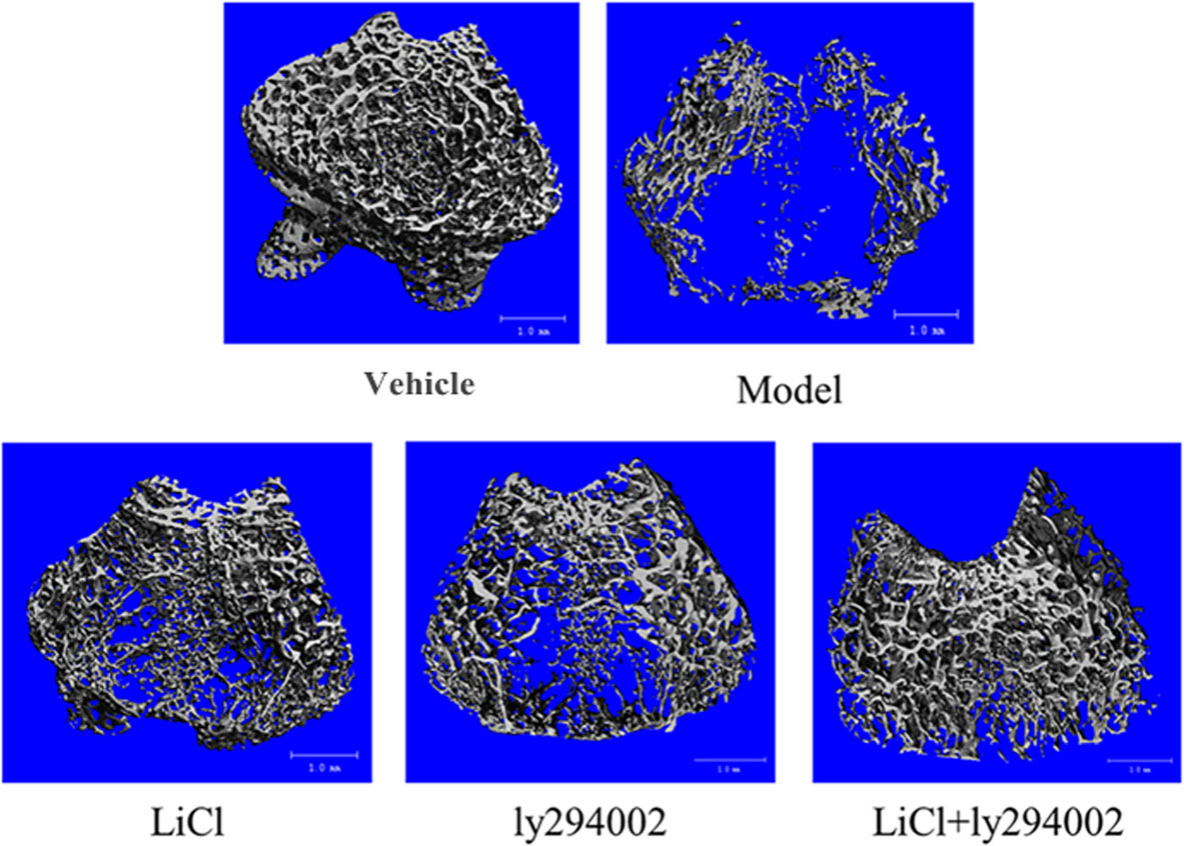
Effects of LiCl, LY294002, and LiCl + LY294002 on the femoral trabecular bone. Control: rats were subjected to sham surgery. OVX: rats were ovariectomized. LiCl: ovariectomized rats were injected with LiCl (10 mM). LY294002: ovariectomized rats were injected with LY294002 (10 μM). LiCl + LY294002: ovariectomized rats were injected with LiCl + LY294002 and LiCl (5 mM) + LY294002 (5 μM). The image shows the 3D trabecular bone architecture within an ROI located under the epiphyseal plate of the femur

In the pathogenesis of osteoporosis, the trabecular bone structure of the distal femur decreases with decreased bone mass. To verify whether the low-dose LiCl and LY294002 combination therapy could be used to treat osteoporosis, we performed a μ-CT analysis of the distal femoral cancellous region of SD rats, as shown in Fig. 6. The trabecular bone in the distal femur decreased significantly after ovariectomy; however, the trabecular bone structure increased significantly after the injection of LiCl or LY294002 alone, and these effects were further improved by the combined application of LiCl and LY294002 (Fig. 6a).

Next, a quantitative analysis of the trabecular bone microstructure in the VOI of the distal femur in ovariectomized rats that received the low-dose combination of LiCl and LY294002 is shown in Table 1. The BMD, BV/TV, connectivity density (COD), Tb.N, and Tb.Th within the ROI of the ovariectomized rats were significantly lower than those of the rats in the vehicle group, whereas the bone surface to bone volume ratio (BS/BV), structure model index (SMI), and Tb.Sp were significantly greater. Comparatively, the combined use of LiCl and LY294002 significantly increased BMD, BV/TV, COD, and Tb.N in the same region. These results suggest that the combined use of LiCl and LY294002 at low doses is a more effective bone loss therapy than the use of either of the two compounds individually in SD rats.

**Table 1:** Microarchitectural quantitative analysis results of an ROI in the femur trabecular bone

	Control	OVX	LiCl	Ly294002	LiCl + ly294002
BMD (mg HA/ccm)	651.93 ± 15.5	503.46 ± 14.3**	569.76 ± 13.6**^##^	577.07 ± 15.8**^##^	605.72 ± 14.1**^##□□△△^
BV/TV (%)	0.2734 ± 0.03	0.0384 ± 0.01**	0.0863 ± 0.01**^##^	0.08 ± 0.01**^##^	0.1352 ± 0.02**^##□□△△^
SMI	1.1439 ± 0.1762	2.7716 ± 0.2553**	2.3604 ± 0.231**^##^	2.5083 ± 0.2275**^##^	2.0728 ± 0.1931**^##□△△^
COD (mm^−3^)	170.4669 ± 13.0462	14.1078 ± 1.437**	57.0813 ± 4.1097**^##^	46.9949 ± 3.7431**^##^	81.2214 ± 5.9857**^##□□△△^
BS/BV (mm^−1^)	35.685 ± 1.04	50.2093 ± 3.98**	48.7623 ± 2.6**	48.4133 ± 2.81**	46.5063 ± 2.22**^##^
Tb.N (mm^−1^)	4.7613 ± 0.41	0.9894 ± 0.08**	2.0506 ± 0.17**^##^	1.9949 ± 0.15**^##^	3.5802 ± 0.28**^##□□△△^
Tb.Sp (mm)	0.1952 ± 0.014	1.0327 ± 0.072**	0.4933 ± 0.032**^##^	0.5059 ± 0.041**^##^	0.2699 ± 0.019**^##□□△△^
Tb.Th (mm)	0.0712 ± 0.006	0.0569 ± 0.004**	0.0556 ± 0.004**	0.0581 ± 0.004**	0.0585 ± 0.004**

BMD, mg HA/cm3; BV/TV, %; SMI, a method for determining the plate- or rod-like geometry of trabecular structures; COD, the number of connections in the trabecular network, which is decreased in osteoporosis; BS/BV, 1/mm; Tb.N, 1/mm; Tb.Sp, mm; Tb.Th, mm. The statistical results shown represent the mean ± standard (*n* = 7) vs the control group, *P < 0.05, **P < 0.01; vs the OVX group, ^#^*P* < 0.05, ^##^*P* < 0.01; vs the LiCl group, ^□^*P* < 0.05, ^□□^*P* < 0.01; vs the ly294002 group, ^△^*P* < 0.05, ^△△^*P* < 0.01

## Discussion

Studies have shown that the Wnt signaling pathways play crucial regulatory roles in bone health and illness development and that they could provide new targets for osteoporosis research and therapy [23]. The Wnt/β-catenin signal stimulates osteoblast proliferation, promotes bone marrow mesenchymal stem cells to differentiate into osteoblasts, promotes the expression of *Osx1* by osteoblasts, and simultaneously inhibits fat and cartilage formation [24]. Moreover, Wnt prevents the apoptosis of mature osteoblasts and prolongs their life cycle via β-catenin-dependent and β-catenin-independent pathways [24]. In addition to the effect on osteoblasts, the Wnt/β-catenin signal stimulates the production and secretion of osteoprotegerin to reduce osteoclast differentiation [25]. Recent studies have also shown that the inactivation of β-catenin in osteoclasts can increase osteoclast number and bone resorption, thereby reducing bone mass. Therefore, the Wnt/β-catenin signaling pathway also plays a critical role in osteoclast activity [11]. Under normal physiological conditions, the PI3K/AKT signaling pathway may selectively alter the physiological functions of osteoblasts and osteoclasts. In the AKT signaling pathway, which regulates GSK3β/NFATC1, inhibition of PI3K was found to downregulate NFATC1 expression and affect osteoclast differentiation in mice, thereby impacting bone mass and bone strength [26]. These studies show that AKT-GSK3β-β-catenin-NFATC1 signaling plays a key role in the treatment of osteoporosis.

A conventional GSK3β inhibitor, LiCl, has been shown to stimulate human mesenchymal stem cell (hMSC) proliferation at lower concentrations (1 and 4 mM) [27], while it suppresses hMSC proliferation at higher concentrations (10, 20 and 40 mM) [14] and in mood disorders [28]; however, treatment with LiCl was followed by severe side effects, such as neurological toxicity [29]. Moreover, LiCl is highly toxic at conventional doses, especially among long-term applications, which severely limits its clinical value [30]. Several studies have previously established that a conventional dose of LY294000 (10 μm) [15], a phosphoinositol-3 kinase (PI3K) inhibitor, also has a high toxicity [31]. Therefore, it is commonly believed that combination therapeutic strategies offer both efficacious and safe treatment. This idea is supported by the potential of LiCl to treat Alzheimer’s disease (AD) [32], as a low-dose combination of LiCl and *Momordica charantia* (MC) not only increased the survival rate of mice by reducing hepatotoxicity but also increased the curative effect [30]. Therefore, this study applied a low-dose combination of LiCl and LY294002 to treat osteoporosis and to test whether this method can inhibit osteoclast activity and promote osteoblast activity through inhibition of the AKT/GSK3β/β-catenin/NFATC1 pathway. This approach can be compared with the concept of active screening at the cellular level to identify the effective combinations of medications. This study also aimed to more clearly elucidate the mechanisms of the combined drug treatment. Such an approach not only provides a theoretical basis for the use of combination therapy to treat osteoporosis but also establishes a basis for the treatment of bone metabolic diseases caused by dynamic bone imbalances.

To study whether the low-dose combination of LiCl and LY294002 can inhibit the AKT/GSK3β/β-catenin/NFATC1 signaling pathway and whether the inhibitory effect of the combination therapy is stronger than that of both drugs alone, p-AKT, AKT, p-GSK3β, GSK3β, β-catenin, p-β-catenin, and NFATC1 expression levels were measured in osteoblasts and osteoclasts. The results of this experiment indicate that the effects of the individual LiCl and LY294002 therapies and the low-dose LiCl and LY294002 combination therapy on the AKT/GSK3β/β-catenin/NFATC1 signaling pathway in osteoblasts and osteoclasts were consistent. Moreover, the effect of the low-dose combination of LiCl and LY294002 was significantly greater than that of either compound administered individually. Recent studies have shown that decreased NFATC1 expression in osteoblasts increases the osteogenic activity of osteoblasts, whereas decreased NFATC1 expression in osteoclasts decreases the bone resorption capacity of these cells [33]. These observations indicate that the low-dose combination of LiCl and LY294002 not only promotes bone formation and inhibits bone resorption but could also be more efficacious for the treatment of osteoporosis than either of the two compounds alone.

We next sought to determine whether the low-dose combination therapy of LiCl and LY294002 could promote osteoblast activity and inhibit osteoclast activity and whether the effects were superior to those of either of the two compounds administered alone. To this end, mineralized nodule formation and ALP activity of osteoblasts and osteoclast absorptive capacity and TRACP activity were investigated. Although LY294002 reduced the osteoblast activity to some extent, the ALP activity of osteoblasts increased significantly when this inhibitor was combined with LiCl. Moreover, mineralized nodule formation was significantly enhanced compared with that resulting from the use of LiCl or LY294002 alone. Compared with the individual treatments, the low-dose combination of LiCl and LY294002 significantly decreased TRACP activity and significantly reduced osteoclast bone resorption. Although the doses of LiCl and LY294002 in combination were half the doses used in the individual treatments, the effects of the combined treatment in vitro were significantly greater than those of either compound alone. Because treating osteoporosis is a long-term process that produces toxic side effects, reducing the required dose while still achieving the optimal therapeutic effect is critical. The combination therapy described here is characterized by small doses of drugs that exert the dual effects of inhibiting bone absorption and promoting bone formation. This approach also reduces the dose required for a single drug while maintaining its efficacy, ensuring optimal osteoporosis treatment.

To verify whether the low-dose combination therapy was superior to the individual administration of LiCl or LY294002 and whether it could exert an anti-osteoporotic effect by promoting bone formation and inhibiting bone resorption, the serum levels of BALP, PINP, TRACP-5b, and CTX in SD rats were measured. Hydroxyapatite and bone collagen are important skeletal components. The levels of the metabolic products of hydroxyapatite and bone collagen may indicate whether the bone tissue is developing an imbalance towards bone formation or bone resorption [23]. BALP is the key enzyme in the formation of bone hydroxyapatite, and PINP is secreted by osteoblasts only during the formation of bone collagen [24]. Therefore, the serum levels of BALP and PINP can be used as markers of bone formation. In contrast, the serum contents of TRACP-5b and CTX can be used as markers of bone resorption; TRACP-5b is secreted by mature osteoclasts and plays a key role in the degradation of skeletal hydroxyapatite, while CTX is a fragment of collagen that is degraded during bone resorption [25]. In this study, BALP and PINP serum levels significantly decreased in ovariectomized rats, whereas TRACP-5b and CTX levels significantly increased. These findings indicate that rats were suffering from osteoporosis, which is consistent with the results of studies by Abuohashish et al. and Dönmez et al. [34, 35]. Both LiCl and LY294002 increased the serum levels of BALP and PINP and decreased the serum levels of TRACP-5b and CTX; however, the combined effects of low-dose LiCl and LY294002 were significantly higher than the effects of either of the two compounds individually. These results indicate that the combined application of low-dose LiCl and LY294002 can promote bone formation and inhibit bone resorption and that the low-dose combination of LiCl and LY294002 is significantly more effective in the treatment of osteoporosis than either of the drugs alone.

In this study, a model of osteoporosis was established in ovariectomized rats to investigate the effects of LiCl or LY294002 administered alone or in a low-dose combination on the cancellous bone region of the distal femur. The small bone region of the distal femur was imaged using μ-CT for 60 days after injecting the treatment directly into the distal femoral condyle. This approach is consistent with that used by Bonnelye et al. [36]. The trabecular structure of the distal femur was significantly damaged after ovariectomy. The low-dose combination of LiCl and LY294002 reversed this trabecular bone damage to a greater extent than either compound alone. Next, BMD, BV/TV, SMI, COD, BS/BV, Tb.N, Tb.Sp, and Tb.Th in the distal femur of the SD rats were quantitatively analyzed. BMD, the mineral content in bone tissue, represents the quantity of mineral contained in the bone volume. The BMD in the ROI of the distal femur was used as a direct indicator of the mineral content of trabecular bone. Because decreased trabecular bone quality is the primary factor leading to decreased biomechanical properties of the femur, BMD reflects the overall degree of osteoporosis. The μ-CT results show that both LiCl and LY294002 alone significantly increased the BMD in the distal trabecular region of the femur. Nevertheless, the BMD in the low-dose combination group was significantly higher than that in the LiCl or LY294002 group. This result suggests that the low-dose combination of LiCl and LY294002 can improve the BMD of osteoporotic bone significantly more than either of the two drugs alone. BV/TV reflects the spatial volume fraction of the ROI and the volume fraction of bone tissue; this ratio is significant because it reflects the bone tissue content in the sample. BV/TV, another major microstructural parameter, reflects the overall mechanical properties of trabecular bone. The μ-CT results show that although the BV/TV of the distal femur trabecular bone was significantly increased by LiCl or LY294002 alone, the low-dose combination therapy achieved a significantly greater BV/TV than that achieved by LiCl or LY294002 alone. These results could potentially be explained by the different effects of LiCl and LY294002 on osteoblasts and osteoclasts, respectively. LiCl promotes osteoblast proliferation and inhibits osteoclast proliferation, while LY294002 inhibits osteoblast proliferation and osteoclast differentiation; however, the inhibitory effect of the low-dose combination of LiCl and LY294002 on osteoclast differentiation was greater than that on osteoblast proliferation, as mediated by the AKT/GSK3β/β-catenin/NFATC1 signaling pathway.

The greater the bone tissue surface area, the more space available for cell attachment; however, with age, trabecular bone volume decreases more rapidly than its surface area, which leads to osteoporosis. Next, the BS/BV of the ROI was detected, and the low-dose combination therapy was found to significantly improve the BS/BV compared with the effects of individual treatments. This result indicates that the effect of local administration of the combination therapy on osteoclastic bone resorption in ovariectomized rats was significant and may play a key role in inhibiting osteoclast differentiation. Our study found that cancellous bone with low bone mass and density had higher SMI values and, thus, would be more likely to develop fractures. Compared with the single-dose groups, the low-dose combination group exhibited a significantly reduced SMI in the distal femoral trabecular bone area, suggesting that the low-dose combination of LiCl and LY294002 inhibits trabecular bone deformation and can thereby reduce the risk of fracture and increase bone strength in osteoporotic patients. Tb.Sp is the mean width of the medullary cavity between the trabeculae of the femur. This study revealed that the low-dose combination of LiCl and LY294002 significantly reduced Tb.Sp; this effect was significantly greater than that of the single application of either compound alone. This result suggests that the low-dose combination of LiCl and LY294002 can significantly enhance the skeletal support provided by the trabecular bone. In summary, the therapeutic effects of low-dose combinations of LiCl and LY294002 on osteoporosis are superior to those of the corresponding monotherapies.

## Conclusions

Using ovariectomized rats as a model, this work demonstrated that the AKT/GSK3β/β-catenin/NFATC1 signaling pathway is a target in osteoporosis treatment. As a proof of concept, the data support that the combination of a low dose of LiCl and LY294002 has the potential to be a more effective therapeutic strategy for the treatment of osteoporosis.

## Data Availability

All data generated or analyzed during this study are included in this published article.

## Abbreviations

ALP: Alkaline phosphatase
BMD: Bone mineral density
BS/BV: Bone surface to volume ratio
BV/TV: Bone volume to tissue volume ratio
COD: Connectivity density
CTX: C-terminal telopeptide of type I collagen
FBS: Fetal bovine serum
GSK-3β: Glycogen synthase kinase 3β
HA: Hydroxyapatite
LiCl: Lithium chloride
M-CSF: Macrophage colony-stimulating factor
NFATc1: Nuclear factor of activated T cells c1
PBS: Phosphate-buffered saline
PI3K: Phosphatidylinositol-3-kinase
PS: Penicillin/streptomycin
RANKL: Receptor activator of nuclear factor-κB ligand
ROI: The region of interest
SD: Sprague-Dawley rats
SMI: Structural model index
Tb.N: Trabecular number
Tb.Th: Trabecular thickness
TRACP: Tartrate-resistant acid phosphatase
α-MEM: α-Minimum essential medium
μ-CT: Microcomputed tomography
